# Probiotic *Bifidobacterium animalis subsp. lactis* DS109-B11 ameliorates age-related muscle weakness via AMPK activation

**DOI:** 10.1038/s41598-026-48725-7

**Published:** 2026-04-12

**Authors:** Jae Won Yang, Min Ju Kim, Heeyeon Jeong, Seongwan Kim, Doo-Sang Park, Yong Ryoul Yang, Kwang-Pyo Lee

**Affiliations:** 1 Aging Convergence Research Center, Korea Research Institute of Bioscience and Biotechnology, Daejeon, 34141 Republic of Korea; 2 Biological Resource Center, Korean Collection for Type Cultures, Korea Research Institute of Bioscience and Biotechnology, Jeongeup, 56212 Republic of Korea; 3 Department of Biomolecular Science, KRIBB School of Bioscience, Korea University of Science and Technology, Daejeon, 34113 Republic of Korea

**Keywords:** Cell biology, Diseases, Microbiology, Molecular biology, Physiology

## Abstract

Sarcopenia, the age-related loss of skeletal muscle mass and function, represents a growing health burden with limited therapeutic options. Given the emerging roles of the gut–muscle axis and AMP-activated protein kinase (AMPK) in muscle homeostasis, we sought to identify gut-derived microbial strains that enhance muscle function via AMPK activation. We identified *Bifidobacterium animalis subsp. lactis* DS109-B11 as a potent AMPK activator. DS109-B11 microbial culture supernatant (MCS) increased AMPK phosphorylation during C2C12 myoblast differentiation, enhanced myogenic differentiation, and mitigated dexamethasone-induced myotube atrophy in vitro. In aged mice, oral administration of live DS109-B11 improved grip strength and motor performance and increased myofiber cross-sectional area, accompanied by elevated AMPK phosphorylation, upregulated mitochondrial and oxidative phosphorylation genes, and downregulated atrophy- and inflammation-related genes in skeletal muscle. In a botulinum toxin–induced neurogenic atrophy model, DS109-B11 treatment partially preserved tibialis anterior muscle mass, improved myofiber cross-sectional area, and suppressed atrophy-related gene expression. These findings identify DS109-B11 as an AMPK-activating probiotic strain that beneficially modulates skeletal muscle differentiation, enhances resilience to catabolic stress, and improves muscle function in vivo.

**Supplementary Information:**

The online version contains supplementary material available at 10.1038/s41598-026-48725-7.

## Introduction

Sarcopenia is a progressive, age-related loss of skeletal muscle mass and function, a major public health concern associated with decreased mobility, frailty, and an increased risk of falls and mortality^1–3^. While exercise and nutritional interventions are key to managing sarcopenia^4^, effective pharmacological treatments remain limited^5,6^. Recent research has shed light on the critical role of the gut-muscle axis, an emerging concept where the gut microbiome and its metabolites can profoundly influence systemic physiology and skeletal muscle homeostasis^7,8^. The intricate crosstalk between the gut and skeletal muscle suggests that modulating the gut microbiome could be a powerful approach to combat age-related muscle decline.

The AMP-activated protein kinase (AMPK) signaling pathway is a central energy sensor that acts as a conserved regulator of cellular metabolism and function across various tissues, including skeletal muscle^9,10^. Its activation is known to promote beneficial physiological processes, such as enhancing mitochondrial biogenesis and glucose uptake^11,12^, while inhibiting catabolic pathways that lead to muscle atrophy^13,14^. Due to its pivotal role in regulating muscle health and its link to longevity^15–17^, AMPK has emerged as a highly promising therapeutic target for combating sarcopenia^18–20^. A growing body of evidence has demonstrated a clear link between microbial-derived factors and AMPK activation. For instance, specific probiotic bacterial strains like *Lactobacillus plantarum* HY7715^21^ and *Lactobacillus rhamnosus* JY02^22^ have been shown to ameliorate sarcopenia by improving skeletal muscle mass and function. Further, a recent landmark study identified lithocholic acid (LCA), a secondary bile acid produced by the gut microbiota, as a potent AMPK activator that mimics the anti-aging effects of caloric restriction and significantly improves muscle function^23^. These findings provide a strong precedent for the therapeutic potential of microbial-derived modulators of the AMPK pathway in the context of sarcopenia.

In this study, we conducted a screening of culture supernatants from various gut microbial strains using C2C12 myotubes and identified a novel strain, DS109-B11, whose culture supernatant potently activates AMPK. Subsequent in vivo studies in aged mice demonstrated that administration of live DS109-B11 bacteria significantly improved muscle function and attenuated muscle atrophy. Our results highlight the potential of targeting the gut microbiome to develop novel strategies for the prevention and treatment of age-related muscle decline.

## Results

### DS109-B11 MCS enhances myogenic differentiation of C2C12 cells through AMPK activation

**Fig. 1:**
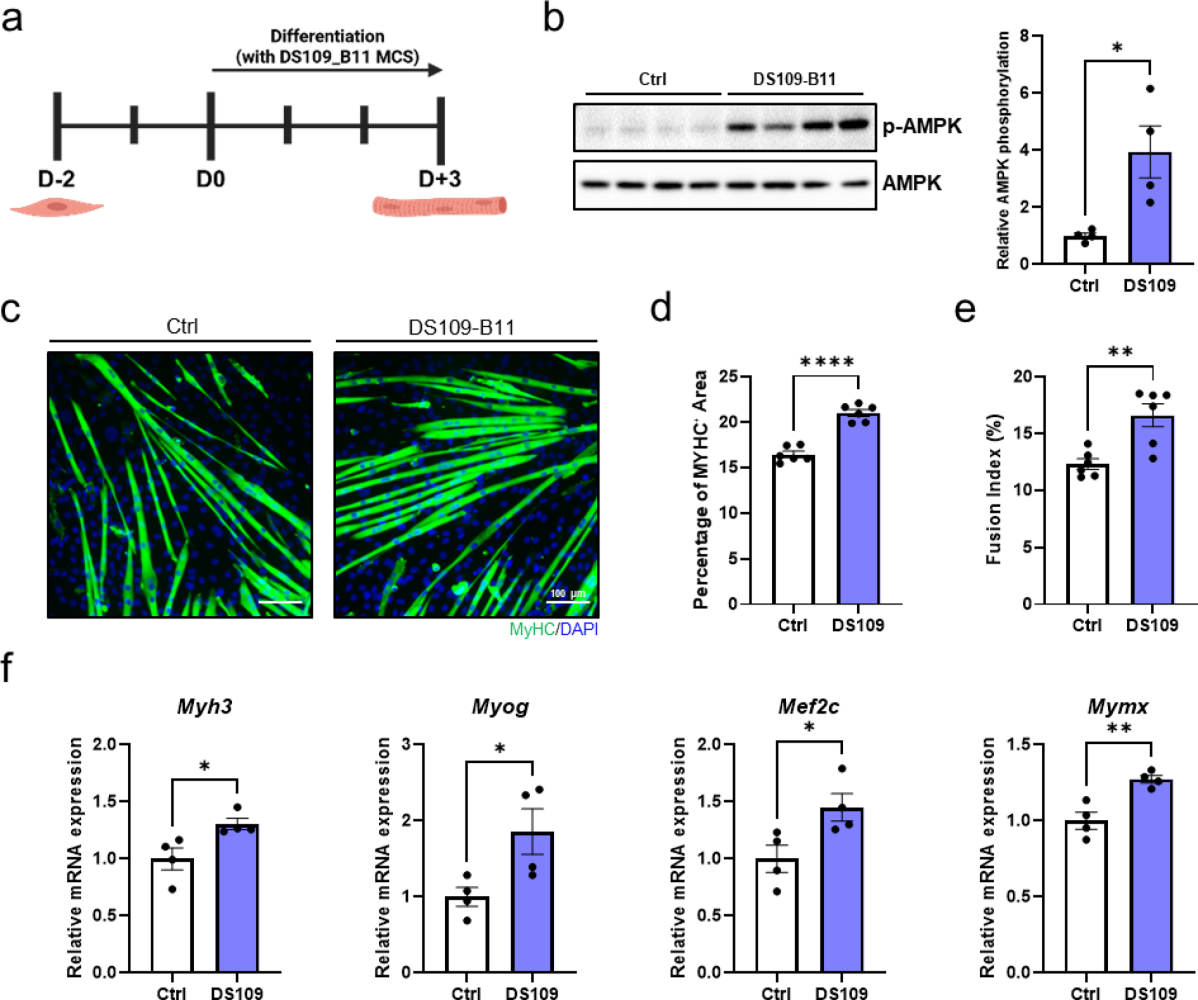
DS109-B11 promotes AMPK activation and myogenic differentiation in C2C12 cells. **a** Experimental scheme for DS109-B11 (DS109) treatment during C2C12 myogenic differentiation. **b** Representative immunoblots of phosphorylated AMPK (p-AMPK) and total AMPK in C2C12 myotubes differentiated with or without DS109 with densitometric analysis (*n* = 4 independent experiments). p-AMPK levels were normalized to total AMPK and are presented as arbitrary units (a.u.). Blots were cropped for clarity, and uncropped blots are shown in Supplementary Figure S4. **c** Representative immunofluorescence images of MyHC-positive (MyHC⁺) C2C12 myotubes differentiated with or without DS109. Scale bars, 100 μm. **d** Quantification of MyHC⁺ area and **e** fusion index of C2C12 myotubes (*n* = 6 independent experiments). **f** qRT-PCR analysis of relative mRNA expression of the differentiation markers *Myh3* and *Myog* and the fusion-related genes *Mef2c* and *Mymx* in C2C12 myotubes (*n* = 4 independent experiments). Gene expression levels were normalized to *36B4* expression. Data are shown as mean ± SEM *P < 0.05, **P < 0.01, ***P < 0.001, ****P < 0.0001. Statistical significance was assessed by Student’s t-test (for b, d, e and f).

DS109-B11 was selected among 150 microbial culture supernatants (MCSs) through a functional screen based on its ability to induce AMPK phosphorylation in C2C12 myoblasts (Supplementary Fig. S1 and Table S1). To characterize the functional effects of the novel strain *Bifidobacterium animalis* subsp. *lactis* DS109-B11, we induced differentiation of C2C12 myoblasts in culture medium supplemented with DS109-B11 microbial culture supernatant (Fig. 1a). C2C12 myoblasts were seeded and subsequently switched to differentiation medium containing DS109-B11 MCS at the onset of differentiation, and cells were harvested or stained after 3 days of differentiation. Western blot analysis revealed that C2C12 myotubes differentiated in the presence of DS109-B11 MCS exhibited markedly elevated AMPK phosphorylation compared with vehicle-treated controls, with an approximately 3.95-fold higher p-AMPK/AMPK ratio (Fig. 1b). Compared to vehicle-treated controls, C2C12 myotubes differentiated with DS109-B11 MCS showed significantly larger myosin heavy chain (MyHC)-positive myotube areas, indicating that DS109-B11 MCS enhances myogenic differentiation. In addition, the fusion index was increased, with MyHC⁺ area and fusion index elevated by approximately 1.28-fold and 1.35-fold, respectively (Fig. 1c–e), reflecting more extensive myoblast fusion. Consistent with the promotion of myogenic differentiation and fusion, qRT-PCR analysis of relative mRNA expression demonstrated increased levels of the differentiation markers *Myh3* and *Myog*^24^ and the fusion-related genes *Mef2c*^25^ and *Mymx*^26^ in C2C12 myotubes differentiated with DS109-B11 MCS compared with controls (Fig. 1f). These findings collectively demonstrated that DS109-B11 MCS promote myogenic differentiation in C2C12 cells through AMPK activation and coordinated enhancement of myoblast fusion.

### DS109-B11 MCS mitigates dexamethasone-induced atrophic responses in C2C12 myotubes

**Fig. 2:**
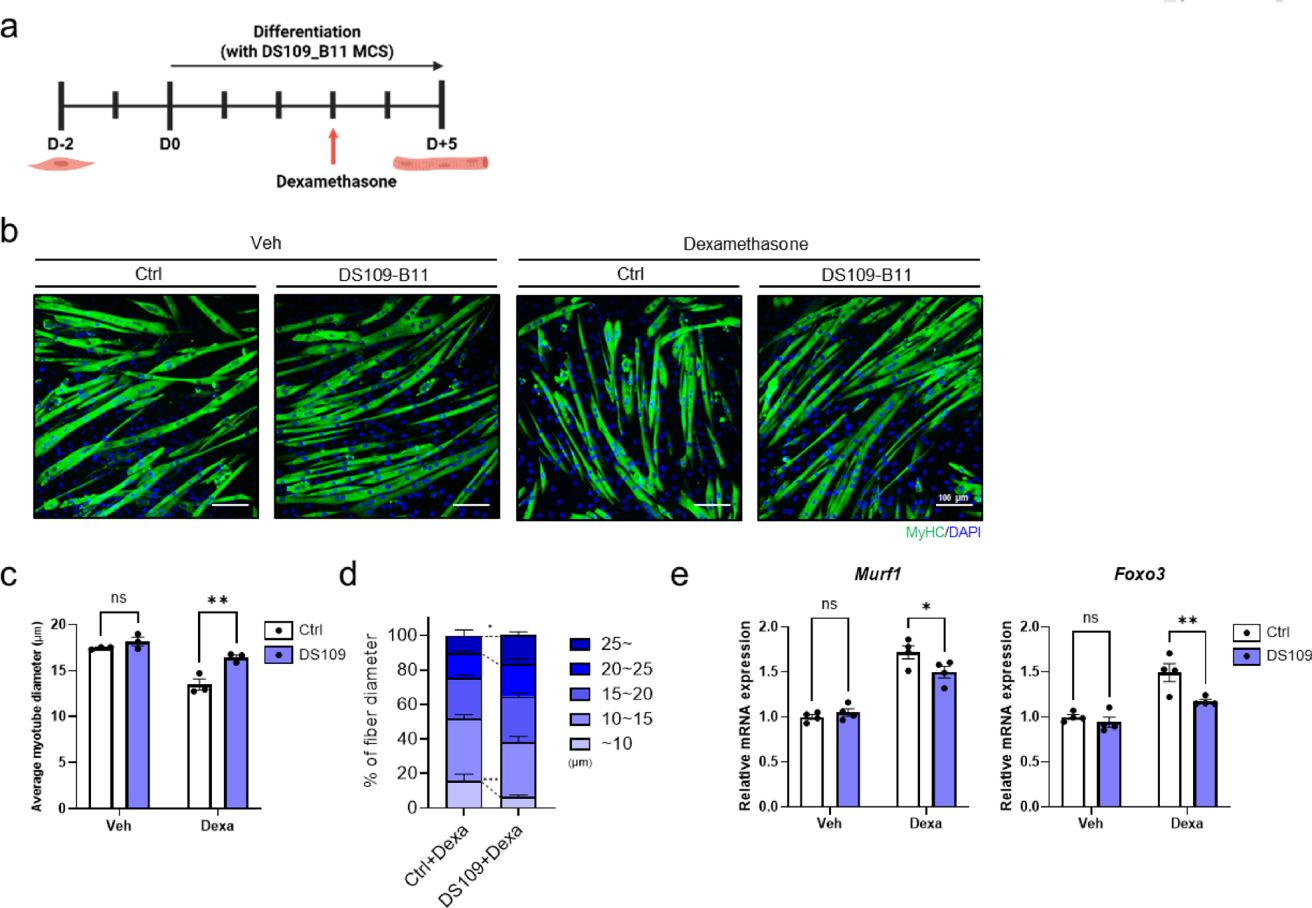
DS109-B11 treatment modulates dexamethasone-induced atrophic responses in C2C12 myotubes. **a** Experimental scheme for dexamethasone-induced atrophy in differentiated C2C12 myotubes with or without DS109-B11 (DS109) treatment. **b** Representative immunofluorescence images of MyHC-positive (MyHC⁺) C2C12 myotubes treated with vehicle or dexamethasone (Dex) in the presence or absence of DS109. Scale bars, 100 μm. Quantification of myotube diameter using ImageJ, showing **c** mean diameter and **d** diameter distribution (*n* = 3 independent experiments). **e** qRT-PCR analysis of relative mRNA expression of the atrophy-related gene *Murf1* in C2C12 myotubes (*n* = 4 independent experiments). Gene expression levels were normalized to *36B4* expression. Data are shown as mean ± SEM. *P < 0.05, **P < 0.01, ***P < 0.001, ****P < 0.0001. Statistical significance was assessed by two-way ANOVA with Šídák’s post hoc test (for c, d and e).

While DS109-B11 MCS promoted myogenic differentiation, its potential to modulate atrophic responses remained unclear. Therefore, we employed a dexamethasone (Dex)-induced atrophy model in C2C12 myotubes^27^, a well-established in vitro system for studying muscle wasting. C2C12 myoblasts were differentiated for 5 days in differentiation medium with or without DS109-B11 microbial culture supernatant (MCS). Dex (100 nM) or vehicle was added on day 3 of differentiation and maintained for an additional 48 h to induce atrophy or maintain basal conditions, respectively (Fig. 2a). C2C12 myotubes under each condition were visualized by MyHC immunofluorescence staining (Fig. 2b), and myotube diameter was quantified to evaluate the effects of DS109-B11 MCS within vehicle- and Dex-treated conditions. In the absence of Dex (vehicle), DS109-B11 MCS did not markedly alter myotube diameter, whereas under Dex treatment, DS109-B11 MCS partially preserved myotube size, resulting in significantly larger mean diameters in Dex + DS109-B11 MCS–treated myotubes compared with Dex-only myotubes (Fig. 2c). In Dex-treated myotubes, DS109-B11 MCS also reduced the proportion of small-diameter myotubes (< 10 μm) and increased the proportion of large-diameter myotubes (≥ 25 μm) compared with Dex alone (Fig. 2d). Consistent with these morphological effects, qRT-PCR analysis of relative mRNA expression revealed that the Dex-induced upregulation of the atrophy-related genes *Murf1*^27^ and *Foxo3*^28^ was attenuated in DS109-B11 MCS–treated myotubes compared with Dex treated controls (Fig. 2e). Collectively, these findings indicate that DS109-B11 MCS partially mitigates Dex-induced atrophic responses in C2C12 myotubes by preserving myotube diameter and limiting the induction of the atrophy-related gene *Murf1* under catabolic conditions.

### DS109-B11 improves muscle function and restores muscle fiber size in aged mice

**Fig. 3:**
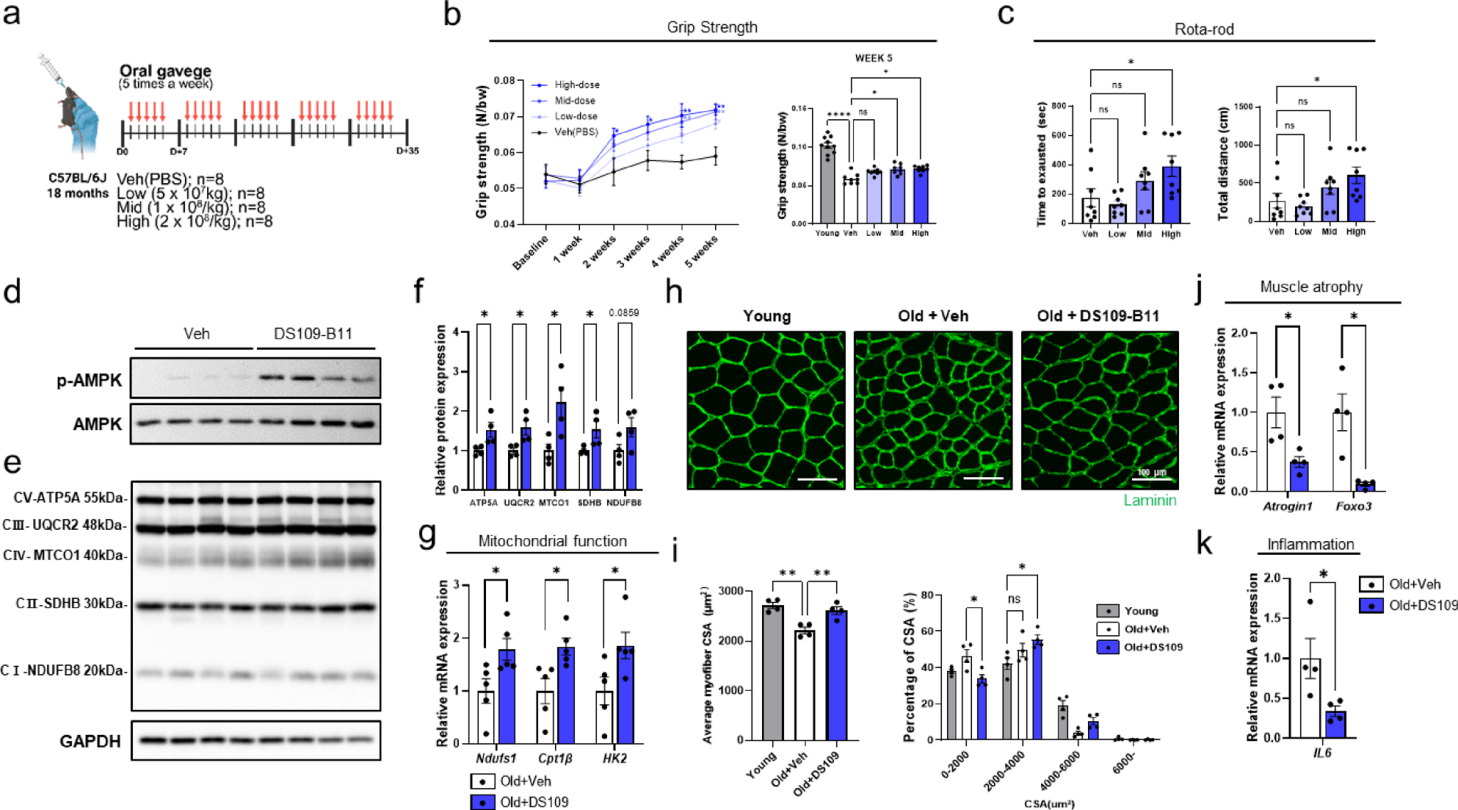
Oral administration of live DS109-B11 bacteria improves muscle function and increases CSA in aged mice. **a** Experimental scheme for oral administration of DS109-B11 live bacteria in aged mice over a 5-week period. **b** Grip strength performance of aged mice treated with vehicle or DS109-B11 live bacteria at low (5 × 10^7^ CFU/kg), mid (1 × 10^8^ CFU/kg), or high (2 × 10^8^ CFU/kg) doses (*n* = 8 per group), showing (left) changes in grip strength from baseline to week 5 and (right) grip strength at week 5. **c** Rota-rod test results in the same aged mouse groups shown in (b) (*n* = 8 per group). **d** Representative immunoblots of p-AMPK, AMPK proteins in tibialis anterior (TA) muscles from aged mice (*n* = 4 mice per group), Blots were cropped for clarity, and uncropped blots are shown in Supplementary Figure S5. **e**, **f** Representative immunoblots of OXPHOS complex proteins soleus muscle from aged mice (*n* = 4 mice per group), and densitometric quantification of OXPHOS levels. Blots were cropped for clarity, and uncropped blots are shown in Supplementary Figure S5. **g** qRT-PCR analysis of mRNA expression of the mitochondrial and OXPHOS-related genes *Ndufs1*, *Cpt1b* and *Hk2* (*n* = 5 mice per group) in soleus muscles. Gene expression levels were normalized to *Gapdh* expression. **h**, **i** Representative images of muscle fiber cross-sectional area (CSA) in TA muscles and quantification of mean CSA and distribution (quantified Using ImageJ) (*n* = 4 mice per group). Scale bars, 100 μm. **j**, **k** qRT-PCR analysis of mRNA expression of the muscle atrophy-related genes *Atrogin1* and *Foxo3* (*n* = 4 mice per group), and the inflammatory marker *Il6* (*n* = 4 mice per group) in TA muscles. Gene expression levels were normalized to *Gapdh* expression. Data are shown as mean ± SEM. *P < 0.05, **P < 0.01, ***P < 0.001, ****P < 0.0001. Statistical significance was assessed by Student’s t-test (for f, g, j and k) or one-way ANOVA with Šídák’s post hoc test (for b, c and i).

Based on our in vitro findings in C2C12 myotubes, we hypothesized that oral administration of live DS109-B11 bacteria would improve muscle function in aged mice. To test this hypothesis, 11-week-old young mice and 18-month-old aged mice were administered vehicle or three doses of DS109-B11 (low: 5 × 10^7 CFU/kg, mid: 1 × 10^8 CFU/kg, high: 2 × 10^8 CFU/kg) by oral gavage once daily, 5 days per week, for 5 weeks (Fig. 3a). Grip strength was measured weekly during the treatment period to assess changes in muscle performance. In aged mice, DS109-B11 treatment increased grip strength in a dose-dependent manner during the early phase of treatment, and the mid- and high-dose groups maintained higher grip strength than vehicle-treated aged mice throughout the 5-week period. Compared with young mice, DS109-B11 partially restored grip strength in aged mice but did not fully normalize age-related reductions in muscle function (Fig. 3b). After the final week of treatment, motor coordination and endurance were evaluated using the rotarod test^29^. Aged mice treated with high-dose DS109-B11 exhibited a significantly longer latency to fall and greater running distance than vehicle-treated aged mice, indicating improved motor performance (Fig. 3c). In addition, we also found that oral administration of DS109-B11 MCS ameliorated muscle weakness in aged mice, showing enhanced grip strength and endurance capacity (Supplementary Fig. S2). Collectively, these functional measurements indicate that DS109-B11 enhances muscle performance in aged mice.

To explore the molecular changes associated with these functional improvements, we examined AMPK signaling and mitochondrial metabolic pathways in skeletal muscle. Immunoblot analysis showed that DS109-B11 treatment increased AMPK phosphorylation without altering total AMPK levels (Fig. 3d) and was associated with enhanced mitochondrial oxidative capacity, as indicated by increased levels of OXPHOS complex proteins in tibialis anterior (TA) muscle (Supplementary Fig. S3a). We also observed higher levels of the OXPHOS complex proteins in the soleus muscle (Fig. 3e, f), which is enriched in oxidative fibers and mitochondrial programs^30^. DS109-B11 significantly increased the expression of *Ndufs1*^31^, *Cpt1β*^32^, and *Hk2*^33^ (Fig. 3g), supporting enhanced mitochondrial and oxidative metabolic gene expression programs.

To characterize the structural alterations in skeletal muscle, cross-sectional area (CSA) analysis of TA muscle fibers showed that DS109-B11 increased myofiber CSA in aged mice and partially reversed the age-associated reduction in fiber size (Fig. 3h, i). We next assessed the expression of genes associated with muscle atrophy and inflammation in TA muscle. DS109-B11 significantly reduced the transcriptional expression of *Atrogin1* and *Foxo3* (Fig. 3j), as well as *Il6*^34^ (Fig. 3k), indicating suppression of atrophy- and inflammation-related gene programs. Taken together, these findings indicate that DS109-B11 modulates AMPK signaling in the skeletal muscle of aged mice, which is associated with suppression of atrophy- and inflammation-related gene expression and enhancement of mitochondrial and oxidative metabolic programs, accompanied by improvements in muscle structure.

### DS109-B11 ameliorates BoNT-A–induced muscle atrophy in young mice

**Fig. 4:**
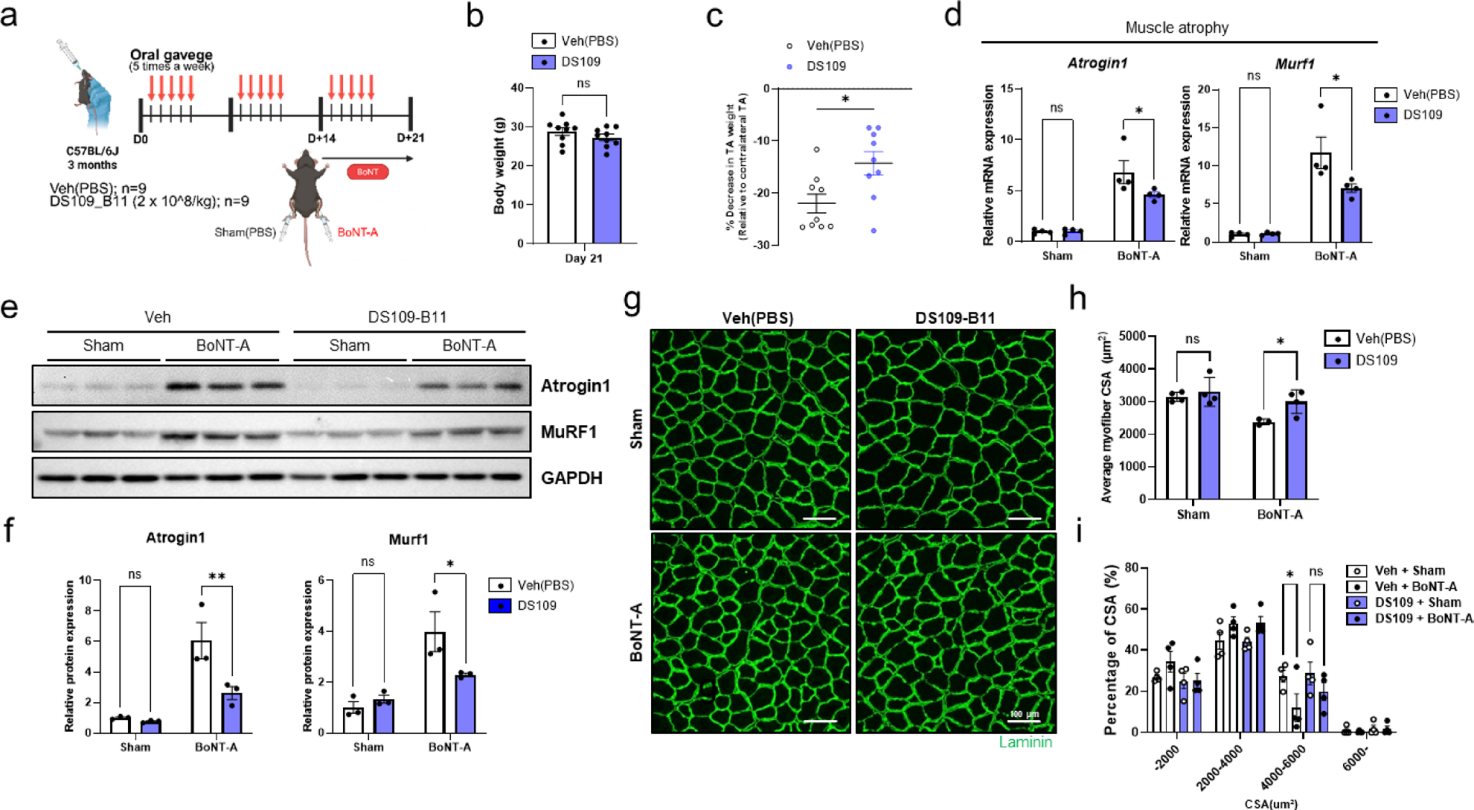
Oral administration of live DS109-B11 bacteria ameliorates BoNT-A-induced muscle atrophy in young mice. **a** Experimental scheme for oral administration of DS109-B11 live bacteria in a BoNT-A-induced muscle atrophy model in young mice. **b** Body weight at day 21 after BoNT-A injection in vehicle- and DS109-B11-treated mice. **c** TA muscle weight of BoNT-A-injected legs normalized to PBS-injected contralateral legs in vehicle- (*n* = 9) and DS109-B11-treated (*n* = 9) mice. **d** qRT-PCR analysis of mRNA expression of the muscle atrophy-related genes *Atrogin1* and *Murf1* in TA muscles, normalized to *Gapdh* expression (*n* = 4 mice per group). **e**, **f** Representative immunoblots of Atrogin1, MuRF1 and GAPDH in tibialis anterior (TA) muscles from a BoNT-A-induced muscle atrophy model (*n* = 4 mice per group), with densitometric quantification. Blots were cropped for clarity, and uncropped blots are shown in Supplementary Figure S6. **g** Representative images of TA muscle fiber cross-sectional area (CSA), **h** quantification of mean CSA, and (i) CSA distribution (quantified using ImageJ) (*n* = 3 mice per group). Scale bars, 100 μm. Data are shown as mean ± SEM. *P < 0.05, **P < 0.01, ***P < 0.001, ****P < 0.0001. Statistical significance was assessed by Student’s t-test (for b and c) or two-way ANOVA with Šídák’s post hoc test (for d, f, h and i).

To further evaluate the anti-atrophic effects of DS109-B11 in vivo, we employed a botulinum toxin type A (BoNT-A)–induced muscle atrophy model, which mimics neurogenic muscle wasting. Because aging is accompanied by progressive neuromuscular junction degeneration and partial denervation, denervation-induced muscle atrophy is considered a relevant model for studying key mechanisms of age-related muscle loss^35^. Previous studies, including our prior report, have shown that BoNT-A administration induces a denervation-like atrophy program in skeletal muscle, characterized by increased expression of the canonical atrophy markers Atrogin1 and MuRF1, along with reductions in muscle mass and contractile function^36–38^. Eleven-week-old C57BL/6J mice were orally administered vehicle or live DS109-B11 bacteria for 14 days. BoNT-A was then injected intramuscularly into the TA muscle, and oral treatment with vehicle or DS109-B11 was continued for an additional 7 days. TA muscles were collected 1 week after BoNT-A administration for analysis (Fig. 4a). DS109-B11 treatment did not alter body weight at the end of the experimental period (Fig. 4b), but it partially preserved TA muscle mass. This effect was reflected by a higher TA muscle weight relative to the contralateral PBS-injected leg in DS109-B11-treated mice compared with vehicle-treated mice following BoNT-A–induced atrophy (Fig. 4c). Consistent with the preservation of muscle mass, qRT-PCR analysis showed that BoNT-A–induced upregulation of the muscle atrophy-related genes *Atrogin1* and *Murf1* was attenuated in DS109-B11-treated TA muscle compared with vehicle-treated BoNT-A–injected muscle (Fig. 4d). To determine whether these transcriptional changes were reflected at the protein level, Western blot analysis further demonstrated that DS109-B11 reduced the BoNT-A-induced expression of Atrogin-1 and MuRF1 proteins in TA muscle, and densitometric quantification confirmed these changes (Fig. 4e, f). Histological analysis further supported these findings. BoNT-A injection caused marked myofiber shrinkage and a shift toward smaller fiber sizes in vehicle-treated muscles, whereas DS109-B11-treated muscles exhibited a partial restoration of fiber size, as confirmed by cross-sectional area (CSA) analysis of TA muscle fibers (Fig. 4g-i). These results suggest that DS109-B11 exerts protective effects against BoNT-A–induced neurogenic muscle atrophy and helps preserve muscle structure in this denervation-associated muscle loss model.

## Discussion

In this study, we identified a microbial strain, DS109-B11, that activates AMPK in skeletal muscle cells and exerts anti-atrophic effects in both in vitro and in vivo models. DS109-B11 was selected through a functional screen based on its ability to induce AMPK phosphorylation in C2C12 myoblasts, and both its microbial culture supernatant and live bacteria improved muscle-related phenotypes under catabolic and aging-associated conditions. These findings support the concept that microbiome-derived factors can engage core exercise-responsive signaling pathways in skeletal muscle, positioning DS109-B11 as a biologically relevant modulator of muscle metabolic adaptation rather than a nonspecific growth-promoting agent.

Our in vitro data demonstrate that DS109-B11 microbial culture supernatant (MCS) enhances myogenic differentiation and selectively confers resistance to catabolic stress without inducing baseline hypertrophy. DS109-B11 MCS increased AMPK phosphorylation during C2C12 differentiation and augmented myogenic fusion, as evidenced by increased MyHC-positive area and fusion index, while myotube diameter and basal expression of the canonical atrogenes Atrogin-1 and Murf1 remained unchanged. This phenotype is consistent with AMPK’s established role as a sensor of energetic stress^9^ and a mediator of exercise-induced muscle adaptation^10^, promoting mitochondrial function, oxidative metabolism, and cellular homeostasis rather than anabolic growth per se^39^. Importantly, the lack of basal atrogene suppression suggests that DS109-B11 does not enforce a constitutive anti-catabolic state but instead enhances adaptive capacity, enabling muscle cells to better respond to stress conditions^40^. This distinction is mechanistically important, as chronic suppression of proteolytic pathways may interfere with normal muscle remodeling and quality control^41,42^. By contrast, AMPK-centered signaling coordinates energy balance, autophagy, and mitochondrial integrity, thereby improving muscle resilience without compromising physiological turnover^14,39,43^. Our findings therefore support a model in which DS109-B11 primes skeletal muscle for improved stress tolerance through AMPK-dependent pathways, selectively attenuating stress-induced atrophy signaling rather than broadly inhibiting protein degradation under basal conditions.

In aged mice, oral administration of DS109-B11 improved muscle function and partially restored myofiber cross-sectional area, accompanied by increased AMPK phosphorylation. Along with these changes, DS109-B11 was associated with reduced transcriptional expression of atrophy- and inflammation-related genes in the TA muscle, and with increased transcriptional programs linked to mitochondrial function and oxidative metabolism in the soleus muscle. This differential response may reflect intrinsic differences in metabolic properties between muscle types: oxidative muscles such as the soleus exhibit higher mitochondrial content and greater oxidative capacity, whereas glycolytic muscles such as the tibialis anterior are more responsive to stress-related transcriptional programs. In this context, AMPK activation may preferentially enhance mitochondrial and oxidative pathways in oxidative muscle, while in glycolytic muscle it may be more closely associated with the suppression of atrophy- and inflammation-related gene expression. Future investigations will be needed to assess protein-level expression of the identified genes.

Notably, these improvements occurred without a significant increase in absolute muscle mass. While this might initially appear limiting, it is mechanistically consistent with an endurance-like adaptation profile driven by AMPK activation^44^, where improvements in muscle quality, metabolic efficiency, and functional performance precede or even outweigh gains in muscle quantity. Given the increasing recognition that muscle strength and function correlate more strongly with clinical outcomes than muscle mass alone^45–47^, these findings underscore the physiological relevance of DS109-B11–mediated adaptations in the context of aging. Consistent with this interpretation, DS109-B11 also conferred partial protection in a BoNT-A–induced neurogenic atrophy model^36^, preserving muscle mass relative to contralateral controls, attenuating induction of Atrogin-1 and Murf1, and improving myofiber cross-sectional area compared with vehicle-treated muscles. The observation that DS109-B11 mitigates muscle wasting across distinct catabolic contexts—systemic aging and denervation-like injury—suggests convergence on shared downstream mechanisms related to energy stress sensing and proteostasis regulation^9,40,43^. Although functional recovery was not assessed in the denervation model, the structural preservation observed supports the notion that DS109-B11 enhances intrinsic muscle robustness independently of neural input.

Several aspects of this work merit further investigation. The specific microbial metabolites responsible for AMPK activation and the corresponding host receptors remain to be identified. While our transcriptional analyses focused on selected metabolic and atrophy-related genes, broader multi-omics approaches may further delineate the signaling networks downstream of DS109-B11. Additionally, longer-term studies and combination strategies incorporating exercise or nutritional interventions may reveal whether sustained or synergistic effects on muscle mass can be achieved over extended periods.

From a translational perspective, the properties of DS109-B11 are particularly noteworthy. Its oral efficacy, microbiome-derived origin^48^, and preferential enhancement of muscle quality rather than overt hypertrophy suggest a favorable safety and applicability profile for aging populations. Unlike pharmacological anabolic agents, which may carry cardiovascular or oncogenic risks^49^, AMPK-centered modulation via microbial or postbiotic approaches may offer a more physiological means^50^ of preserving muscle function. Thus, DS109-B11 may represent a complementary strategy to exercise, especially for individuals with limited mobility, frailty, or reduced exercise tolerance. In summary, this study identifies DS109-B11 as a microbiome-derived modulator of skeletal muscle aging that improves muscle quality and stress resilience through AMPK-centered mechanisms. By selectively enhancing adaptive capacity rather than enforcing anabolic growth, DS109-B11 offers a mechanistically grounded and translationally attractive approach for mitigating muscle wasting in aging and catabolic conditions.

## Methods

### Bacterial culture and preparation of bacterial cell-free supernatants

The 150 bacterial strains comprise 17 species were obtained from the Bio R&D Product program (https://biorp.kribb.re.kr/, Supplementary Table S1) and from the Korean Collection for Type Cultures (KCTC). The strains were cultivated in de Man, Rogosa, and Sharpe (MRS) broth (BD, Sparks, MD, USA) under anaerobic conditions at 37℃ for 24 ~ 48 h to produce stationary phase cultures. The bacterial cultures were centrifuged at 3,000 ×g for 10 min. The resulting microbial culture supernatant (MCS) were heated at 65 °C for 30 min and filtered through 0.22-µm syringe filter, then transferred to a new tube and kept − 70℃ until use. For the in vivo study, the DS109_B11 bacterial strain was produced by Huons N using a standardized production process.

### Cell culture and differentiation

The C2C12 mouse myoblast cell line (CRL–1772, ATCC, female) was cultured in a humidified incubator at 37 °C with 5% CO₂. Cells were grown in Dulbecco’s Modified Eagle’s Medium (DMEM, LM001–05, Welgene) supplemented with 10% fetal bovine serum (FBS, 16000044, Gibco), 100 U/mL penicillin, and 100 µg/mL streptomycin (LS202–02, Welgene). For differentiation experiments, C2C12 myoblasts were seeded at a density of approximately 2 × 10⁴ cells/cm² under identical conditions across all treatment groups. After approximately 2 days, when cells reached 80–90% confluence, differentiation was induced by switching to DMEM containing 2% FBS, penicillin, and streptomycin for 3–5 days. During differentiation, DS109-B11 microbial culture supernatant (MCS) was diluted in fresh differentiation medium to a final concentration of 0.01% (v/v) and then applied to the cells. Control cells received differentiation medium prepared in the same way without MCS. For in vitro screening, C2C12 myoblasts were seeded in 24-well plates and the culture medium was replaced with fresh medium containing 0.01% (v/v) microbial culture supernatants (MCSs). After 12 h of treatment, cells were harvested in RIPA buffer and lysates were subjected to Western blot analysis.

### Dexamethasone-induced myotube atrophy model

To induce myotube atrophy, C2C12 myoblasts were differentiated for 3 days in differentiation medium with or without DS109-B11 microbial culture supernatant (MCS). DS109-B11 MCS was prepared by diluting the supernatant in fresh differentiation medium to a final concentration of 0.01%. After 3 days of differentiation, dexamethasone (Dexa) was added to the differentiation medium at a final concentration of 100 nM and cells were incubated for an additional 48 h. Vehicle-treated cells received the same volume of vehicle in parallel. DS109-B11 MCS treatment was maintained during the Dexa or vehicle exposure period. Cells were then subjected to downstream analyses as indicated, including immunofluorescence staining and gene expression analysis.

### Animal experiments

C57BL/6J male mice were used for all experiments. Young mice (3 months old) and aged mice (18 months old) were obtained from the Laboratory Animal Resource Center (LARC) at KRIBB. Mice were housed in individually ventilated cages maintained at 22–24 °C under a 12 h light/dark cycle with ad libitum access to water and standard chow (2018 S, Teklad). Mice were monitored throughout the study for general health status. At the end of each experiment, all mice were euthanized by cervical dislocation in accordance with institutional guidelines, and the TA and soleus muscles were collected for qRT-PCR analysis, Western blotting, and immunohistochemistry, including muscle fiber cross-sectional area (CSA) measurements. All experiments were conducted in accordance with the ARRIVE guidelines and approved by the Animal Care and Use Committee of KRIBB.

### DS109-B11 supplementation in aged mice

18-month-old mice were orally gavaged with live DS109-B11 at three doses. The low dose was 5 × 10^7^ CFU/kg, the mid dose was 1 × 10^8^ CFU/kg, and the high dose was 2 × 10^8^ CFU/kg. Administration was performed 5 days per week for 5 weeks. PBS was used as the vehicle control for all in vivo treatments. Forelimb grip strength was monitored during the study, and final grip strength and rotarod performance were assessed at week 5.

### BoNT-A–induced muscle atrophy model

In 3-month-old mice, DS109-B11 supplementation was initiated at the high dose (2 × 10^8 CFU/kg) and maintained for 2 weeks before induction of muscle atrophy. Mice were anesthetized with tribromoethanol (Avertin; T48402, Sigma) prepared in 2-methyl-2-butanol (tert-amyl; 152643, Sigma) and administered intraperitoneally at 570 mg/kg. Botulinum toxin A (BoNT-A, Allergan) was then administered to one hind limb (5 U/kg), and DS109-B11 supplementation was continued for an additional 1 week after BoNT-A administration. The contralateral limb received PBS (vehicle). TA muscle mass was compared between the BoNT-A–treated limb and the contralateral vehicle-treated limb within the same animal. PBS was used as the vehicle control for all in vivo treatments.

### Western blotting

Cell and tissue lysates were prepared using radioimmunoprecipitation assay (RIPA) buffer supplemented with protease and phosphatase inhibitors (0.1 mM Na₃VO₄, 1 mM NaF, 1 mM AEBSF, and 5 mg/mL aprotinin). Total protein concentration was measured using bicinchoninic acid (BCA) assays. Samples were then resolved by SDS-PAGE and electrotransferred to nitrocellulose membranes. To minimize nonspecific binding, membranes were incubated for 1 h at room temperature with either 5% non-fat dry milk or 2% bovine serum albumin (BSA) in Tris-buffered saline. Following overnight incubation at 4 °C with primary antibodies diluted 1:1000, membranes were washed and probed for 1 h at room temperature with horseradish peroxidase (HRP)-conjugated secondary antibodies. Protein bands were visualized using a chemiluminescence imaging system. Band intensities were quantified using ImageJ software. Primary antibodies used were anti-AMPKα (Cell Signaling Technology, #2532), anti-phospho-AMPKα (Thr172) (Cell Signaling Technology, #2525), and Total OXPHOS Rodent WB Antibody Cocktail (Invitrogen, 45–8099). GAPDH was detected using a specific antibody produced in our laboratory^36,51–53^. Uncropped immunoblot images are provided in the Supplementary Fig S4-S9.

### RNA isolation and quantitative RT-PCR (qPCR)

Total RNA was extracted from C2C12 myotubes using an easy-BLUE™ Total RNA Extraction Kit according to the manufacturer’s instructions (iNtRON Biotechnology, Seongnam, Korea). RNA quality was assessed using the Agilent 2100 Bioanalyzer (Agilent Technologies, Amstelveen, The Netherlands), and RNA quantification was performed using an ND-2000 Spectrophotometer (Thermo Fisher Scientific, Waltham, MA, USA). Quantitative real–time PCR was performed using a StepOnePlus™ Real-Time PCR System (Applied Biosystems). Each 20 µL reaction contained cDNA, gene-specific primers, and SYBR Green Master Mix (Applied Biosystems). The data were normalized to an internal control gene, with *36B4* used for in vitro experiments and *GAPDH* used for in vivo experiments. Primers sequences are provided in Supplementary Table S4.

### Immunocytochemistry

C2C12 myoblasts were seeded onto sterile glass coverslips placed in 12-well plates and differentiated into myotubes. Following differentiation, myotubes were gently washed with PBS and fixed with 4% paraformaldehyde for 15 min at room temperature. Cells were washed three times with PBS to remove residual fixative and permeabilized with 0.25% Triton X-100 in PBS for 10 min. To minimize background staining, myotubes were blocked for 30 min with 1% BSA in PBS containing 0.1% Tween-20. Cells were then incubated overnight at 4 °C with an anti-MYH primary antibody (SC-376157, Santa Cruz) diluted in blocking buffer. After washing with PBS, myotubes were incubated for 1 h at room temperature with an Alexa Fluor^®^ 488-conjugated secondary antibody (A-21121, Thermo Fisher) diluted in 1% BSA in PBS. Coverslips were washed with PBS and mounted onto glass slides using a mounting medium containing DAPI (H-1200, VECTOR Laboratories). Images were captured under a Nikon Eclipse Ti-U inverted microscope equipped with a Nikon DS-Ri2 camera and NIS–Elements software. For quantitative analyses, three randomly selected fields per sample were imaged under identical acquisition settings. Myotube diameter and MyHC-positive area were quantified using ImageJ (NIH) software. For myotube diameter measurements and fiber diameter distribution analysis, at least 25 myotubes per image were measured (≥ 75 myotubes per sample), and the mean value from the three images was used as a single value for statistical analysis. The fusion index was calculated as the percentage of nuclei located within MyHC-positive myotubes containing ≥ 2 nuclei relative to the total number of nuclei in the field. Nuclei were identified by DAPI staining.

### Immunohistochemistry and Immunofluorescence

Muscle tissues were excised at resting length, embedded in optimal cutting temperature (OCT) compound (4583, Tissue-Tek), rapidly frozen in liquid nitrogen, and stored at − 80 °C. After equilibration to the cryostat temperature, transverse Sect. (10 μm) were prepared from the mid-belly portion using a cryostat (CM1950, Leica) maintained at − 20 °C. Sections were mounted on glass slides (5116–20 F, MUTO, Japan) and stored at − 80 °C until immunostaining. For immunofluorescence staining, consecutive sections were brought to room temperature, air-dried for 30 min, and washed three times in PBS. Sections were then subjected to a combined permeabilization and blocking step in PBS containing 0.4% Triton X-100 for 30 min. Primary antibody against laminin (L9393, Sigma-Aldrich) was diluted in PBS supplemented with 10% goat serum (ab7481, Abcam) and incubated for 2 h at room temperature. After primary incubation, sections were incubated with Alexa Fluor^®^ 488-conjugated secondary antibody (A-21121, Thermo Fisher) diluted in PBS containing 10% goat serum. Sections were washed three times with PBS and mounted using Vectashield mounting medium containing DAPI (H–1200, VECTOR Laboratories). Fluorescence images were collected on a Nikon Eclipse Ti–U inverted microscope fitted with a Nikon DS-Ri2 camera and controlled by NIS-Elements software. Quantitative image analysis was performed using NIS-Elements software. For quantitative analyses, at least five randomly selected images per sample were analyzed, and more than 100 muscle fibers per sample were evaluated.

### Muscle function assessment

Muscle function was assessed using grip strength and rotarod tests. For grip strength, mice were allowed to grasp an angled grid with all four paws, and peak force (N) was measured as the mouse was gently pulled away from the grid until release. Measurements were obtained in three trials per mouse, and the mean value was used for analysis. Motor coordination and balance were evaluated using a rotarod apparatus (ROTA-ROD, B.S Techno Lab Inc., Seoul, Korea) operated at a constant speed of 10 rpm. Each mouse completed three trials, and the longest latency to fall was used for analysis.

### Statistical analysis

All data are shown as mean ± SEM, unless otherwise specified. The Shapiro–Wilk test was used to assess the normality of the distribution. GraphPad Prism (GraphPad Software Inc., USA) was used to conduct the Student’s t-test and non-parametric Mann-Whitney test. One-way ANOVA followed by Šídák’s post hoc test was used for comparisons among multiple groups. Two-way ANOVA followed by Šídák’s post hoc test was applied to evaluate the effects of two independent variables and their interaction. The statistical test used for each analysis is indicated in the corresponding figure legends, and detailed statistical results, including *P* values from t-tests, interaction *P* values from two-way ANOVA, and post hoc *P* values, are provided in Supplementary Table S2 and S3. *P* values less than 0.05 were considered statistically significant. Statistical significance is indicated as *P* < 0.05, *P* < 0.01, *P* < 0.001, and *P* < 0.0001.

## Supplementary Information

Below is the link to the electronic supplementary material.

Supplementary Material 1

## Data Availability

The data that support the findings of this study are available from the corresponding author upon reasonable request.
