# Supplementary material for: Probiotic Bifidobacterium animalis subsp. lactis DS109-B11 ameliorates age-related muscle weakness via AMPK activation
Source: Sci Rep. 2026 Apr 12;16:17017. doi: 10.1038/s41598-026-48725-7 (PMC13230923; doi:10.1038/s41598-026-48725-7)
Supplement: Supplementary file 1 — Supplementary Material 1 [file 41598_2026_48725_MOESM1_ESM.pdf]

Supplementary Figure S1

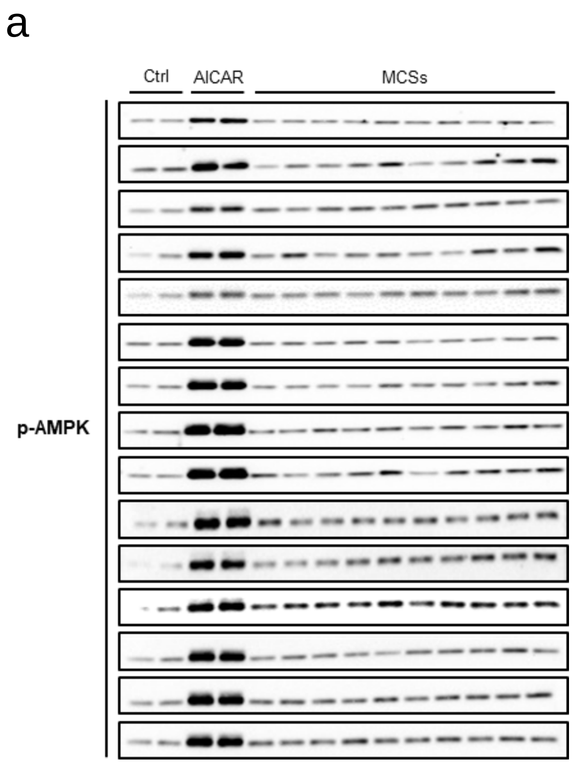

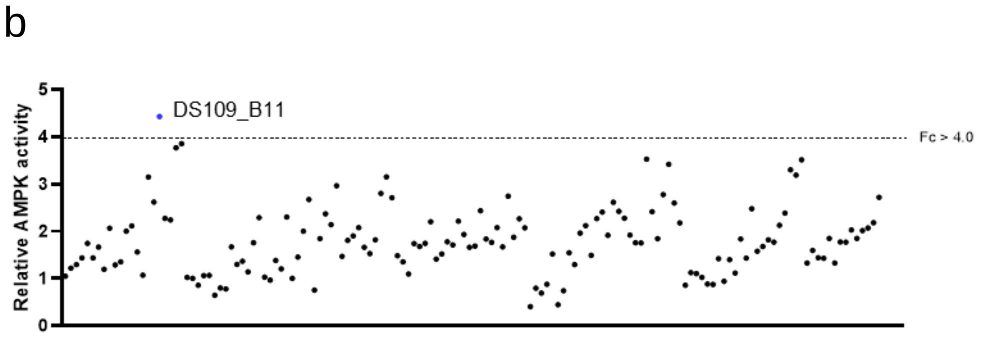

Supplementary Figure S1. Immunoblot screening identifies DS109-B11 MCS as the strongest AMPK-activating candidate. (a) Immunoblot screening of phosphorylated AMPK (p-AMPK) across 150 microbial cultured supernatants (MCSs). Representative blots from the screening are shown. (b) Dot plot of densitometric quantification of p-AMPK signals across the full MCS library, showing that DS109-B11 MCS induced the highest level of AMPK phosphorylation. Values were calculated relative to the control lane within the same blot. Blots were cropped for clarity, and uncropped blots are shown in Supplementary Figure S7.

Supplementary Figure S2

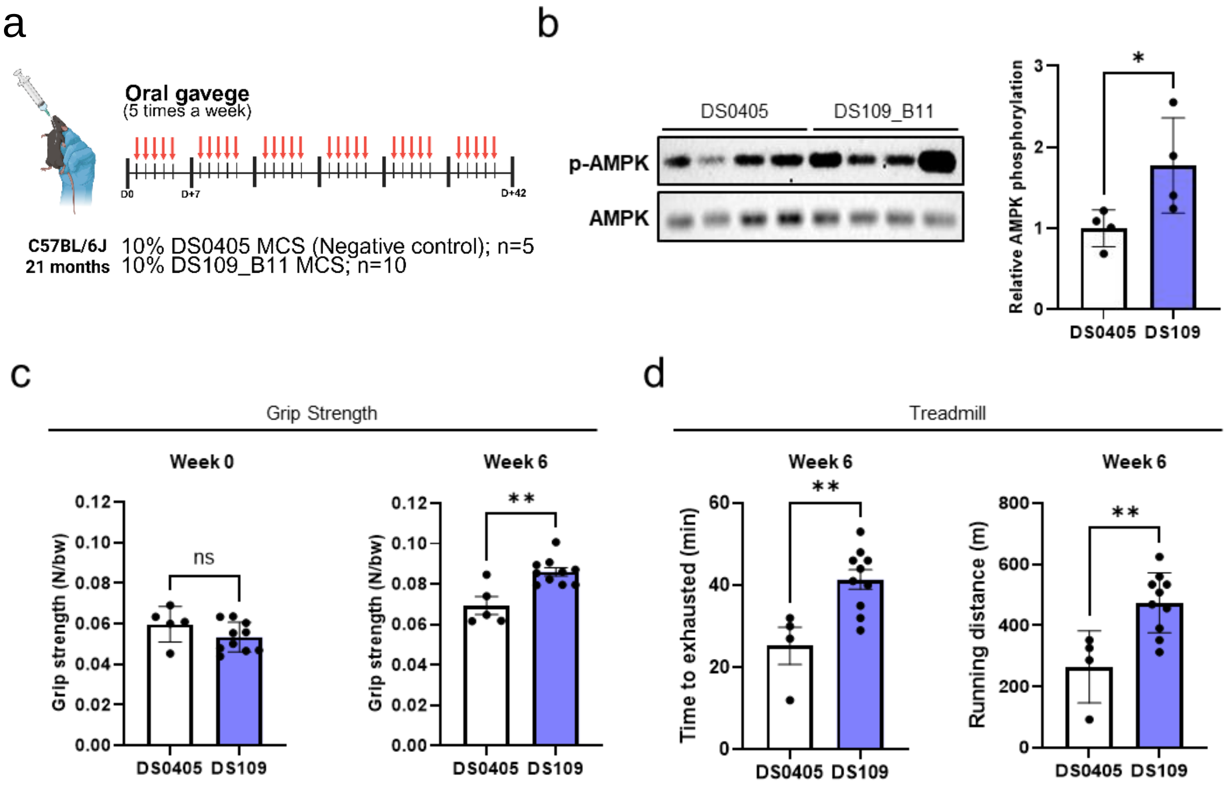

Supplementary Figure S2. Oral administration of DS109-B11 MCS activates AMPK signaling and improves muscle function in aged mice. (a) Experimental scheme for 6-week oral gavage of 10% (v/v) DS109-B11 MCS. DS109-B11 MCS was diluted in PBS and administered to aged mice by oral gavage for 6 weeks. (b) Immunoblot analysis of p-AMPK and total AMPK, with densitometric quantification. Blots were cropped for clarity, and uncropped blots are shown in Supplementary Figure S8. (c) Grip strength test results at week 0 (left) and week 6 (right).(d) Treadmill performance shown as time to exhausted (left) and running distance (right).

Supplementary Figure S3

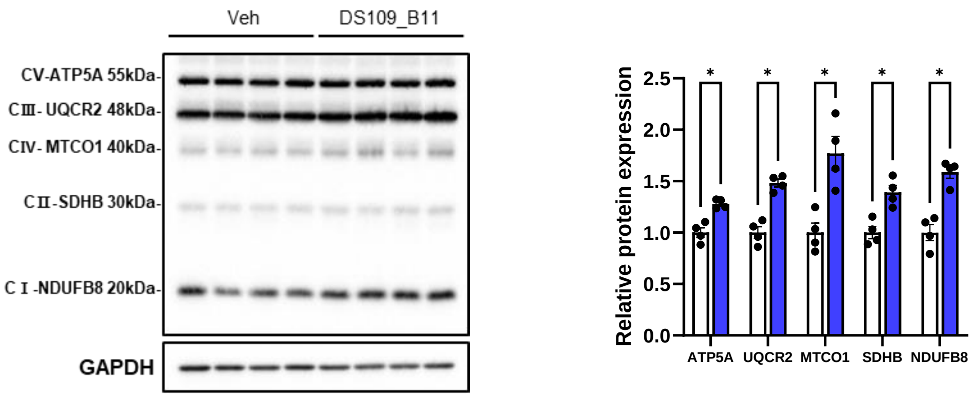

Supplementary Figure S3. DS109-B11 modulates OXPHOS complex proteins in TA muscle of aged mice. Immunoblot analysis of OXPHOS complex proteins in TA muscle of aged mice, with densitometric quantification. Blots were cropped for clarity, and uncropped blots are shown in Supplementary Figure S9.

Supplementary Figure S4

a

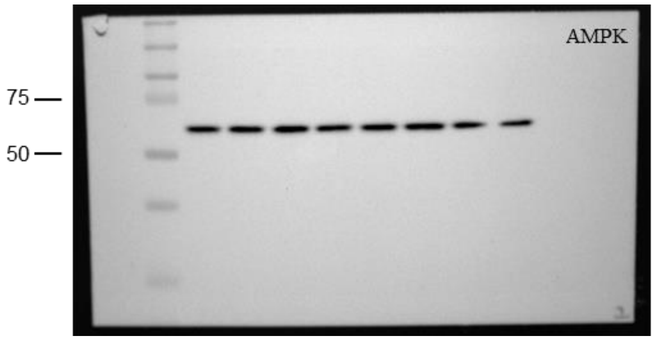

b

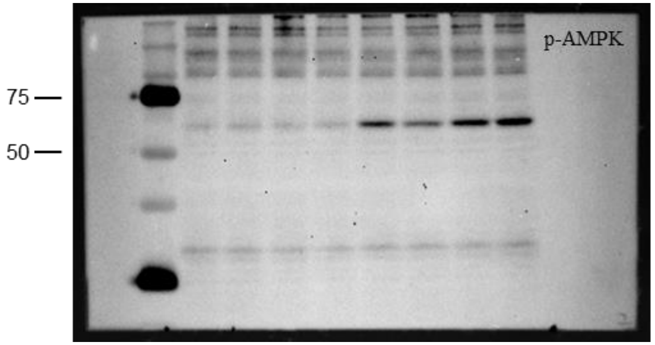

Supplementary Figure S4. Uncropped immunoblots corresponding to Fig. 1b. (a) Uncropped immunoblot for total AMPK in C2C12 myotubes. (b) Uncropped immunoblot for p-AMPK in C2C12 myotubes.

Supplementary Figure S5

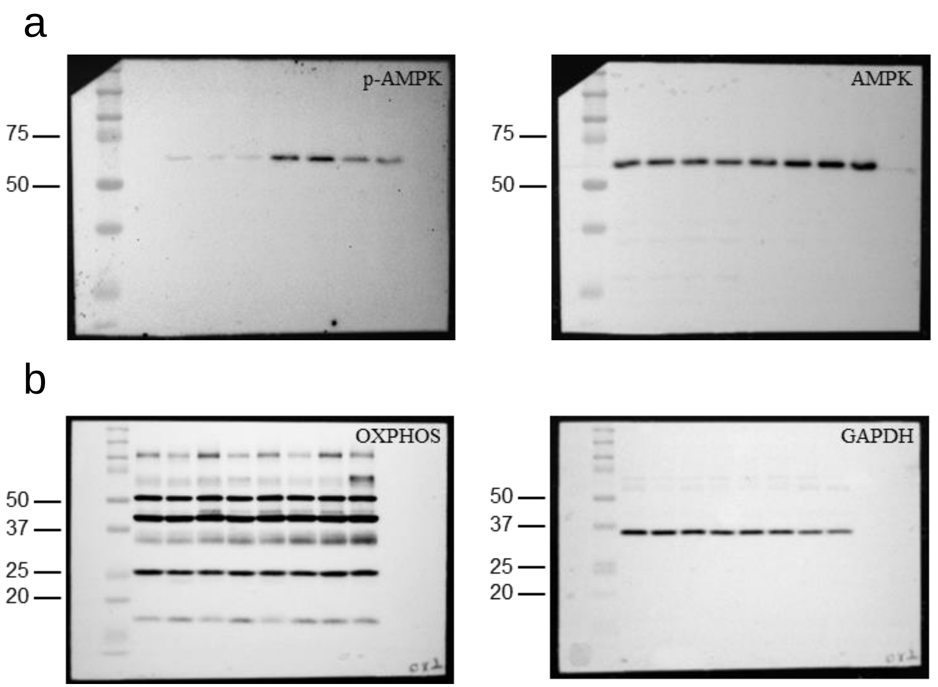

Supplementary Figure S5. Uncropped immunoblots corresponding to Fig. 3d and e. (a) Uncropped immunoblot for phosphorylated AMPK (left) and total AMPK (right) in the TA muscle of aged mice. (b) Uncropped immunoblot for OXPHOS complex proteins and GAPDH in the soleus muscle of aged mice.

Supplementary Figure S6

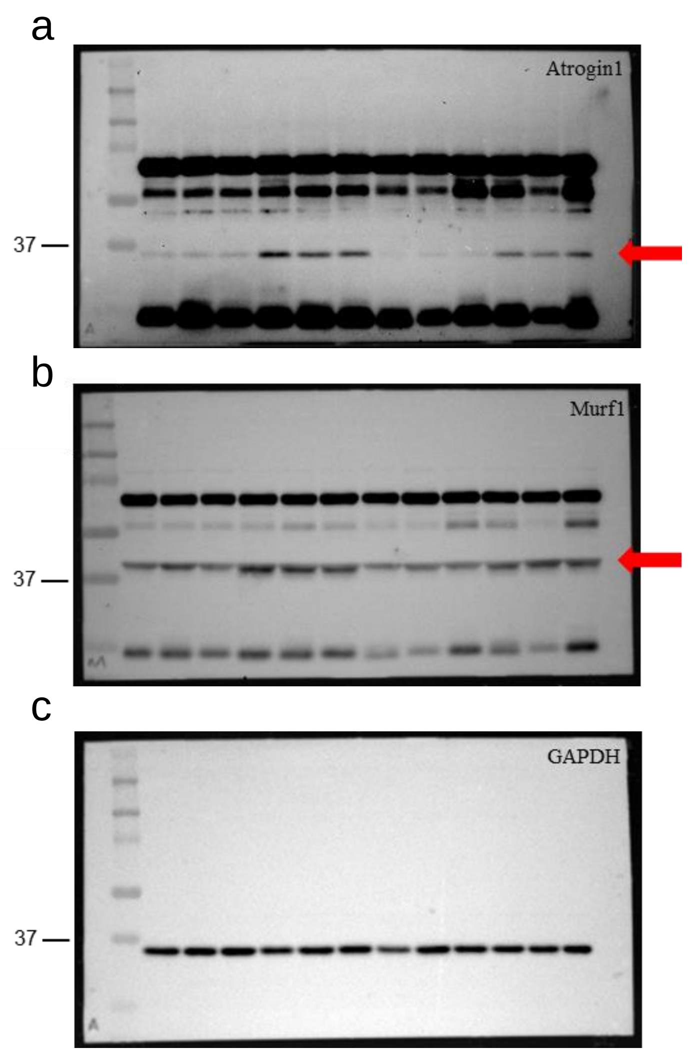

Supplementary Figure S6. Uncropped immunoblots corresponding to Fig. 4e. (a) Uncropped immunoblot for Atrogin1 in TA muscle. (b) Uncropped immunoblot for MuRF1 in TA muscle. (c) Uncropped immunoblot for GAPDH in TA muscle.

a

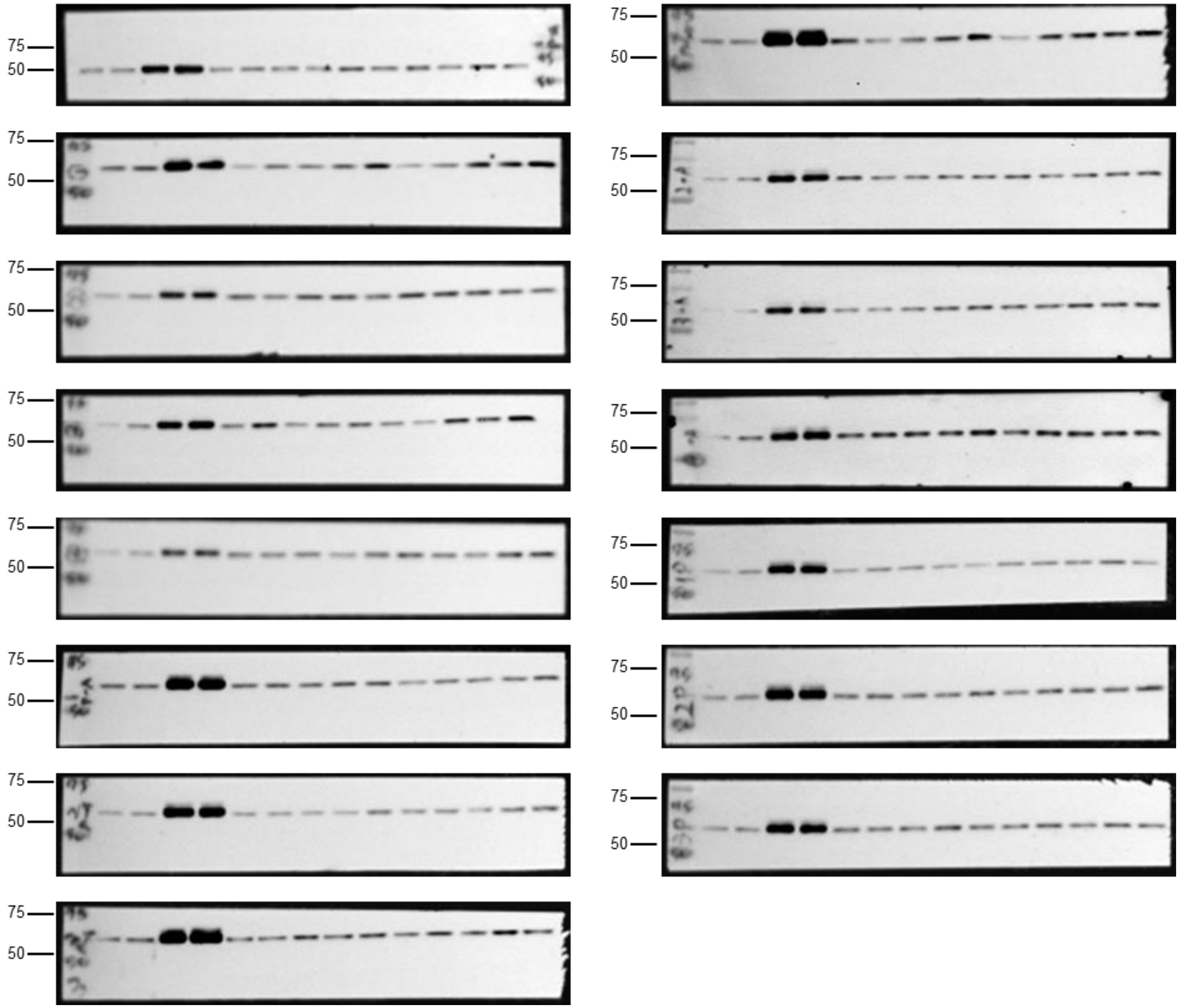

Supplementary Figure S7. Uncropped immunoblots corresponding to Supplementary Figure S1

Supplementary Figure S8

a

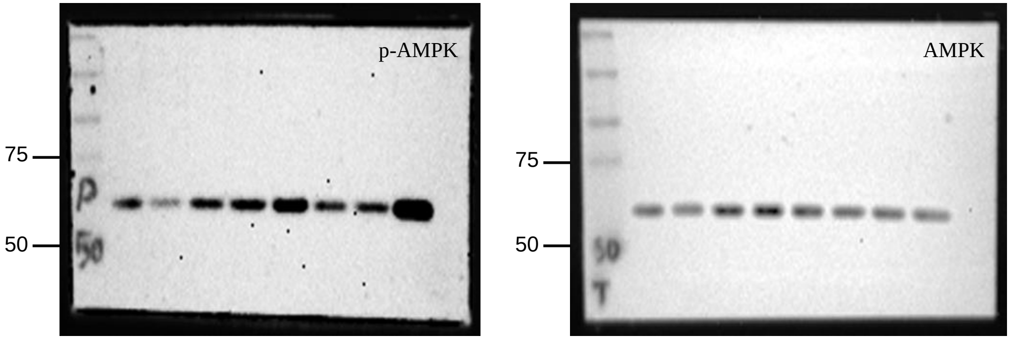

Supplementary Figure S8. Uncropped immunoblots corresponding to Supplementary Figure S2b. Uncropped immunoblot for p-AMPK and AMPK in the TA muscle of aged mice.

Supplementary Figure S9

a

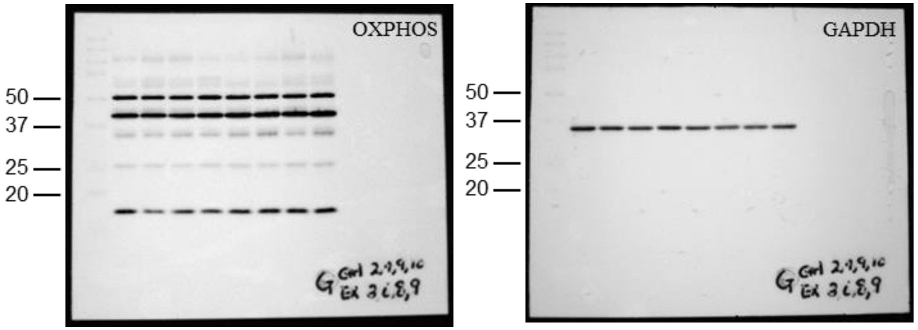

Supplementary Figure S9. Uncropped immunoblots corresponding to Supplementary Figure S3. Uncropped immunoblot for OXPHOS complex proteins and GAPDH in the TA muscle of aged mice.

Supplementary Table S1

Species	Number
Bifidobacterium animalis	4
Bifidobacterium animalis subsp. Lactis	3
Bifidobacterium bifidum	7
Bifidobacterium breve	11
Bifidobacterium longum	12
Bifidobacterium longum subsp. infantis	13
Enterococcus faecalis	3
Lactobacillus acidophilus	3
Lactobacillus fermentum	13
Lactobacillus gasseri	20
Lactobacillus paracasei	18
Lactobacillus plantarum	12
Lactobacillus reuteri	6
Lactobacillus rhamnosus	17
Lactobacillus salivarius	1
Pediococcus pentosaceus	6
Streptococcus salivarius subsp. Salivarius	1

Supplementary Table S1. List of bacterial species and strain counts used for *in vitro* screening.

Supplementary Table S2

Supplementary Table S2. Detailed statistical results for Figure 1–4.

Figure	Statistical test	Post-hoc or t-test (P value)	Interaction P value
1b	Unpaired Student's *t*-test	1b (0.0182)	-
1d,e	Unpaired Student's *t*-test	1d (<0.0001)<br>1e (0.0033)	-
1f	Unpaired Student's *t*-test	*Myh3* (0.0306)<br>*MyoG* (0.0383)<br>*Mef2c* (0.0379)<br>*Mymx* (0.0045)	-
2c	Two-way ANOVA + Šídák	Ctrl vs DS109<br>Veh (0.4694), Dexa (0.0020)	DS109_B11 x Dexa<br>(0.0256)
2d	Two-way ANOVA + Šídák	Ctrl+Dexa vs DS109+Dexa<br>~10 (0.0010), 25~ (0.0298)	DS109_B11 x Dexa<br>(0.0001)
2e	Two-way ANOVA + Šídák	*Murf1*, Ctrl vs DS109<br>Veh (0.7729), Dexa (0.0309)<br><br>*Foxo3*, Ctrl vs DS-109<br>Veh (0.7618), Dexa (0.0055)	DS109_B11 x Dexa<br>*Murf1* (0.0302)<br><br>*Foxo3* (0.0500)
3b,c	one-way ANOVA + Šídák	Grip<br>Veh-Mid (0.0215) Veh vs High (0.0144)<br>Rota(sec) Veh vs High (0.0337)<br>Rota(cm) Veh vs High (0.0329)	-
3f	Unpaired Student's *t*-test	Atp5a (0.0365)<br>Uqcr2 (0.0254)<br>Mtco1 (0.0299)<br>Sdhb (0.0475)<br>Ndufb88 (0.0859)	-
3g	Unpaired Student's *t*-test	*Ndufs1* (0.0339)<br>*Cpt1b* (0.0188)<br>*Hk2* (0.0451)	-
3i	one-way ANOVA + Šídák	Average CSA<br>Young vs Old+Veh (0.0016)<br>Old+Veh vs Old+DS109 (0.0071)<br><br>Distribution -2000<br>Old+Veh vs Old+DS109 (0.0179)<br>Distribution 2000-4000<br>Young vs Old+Veh (0.3693)<br>Young vs Old+DS109 (0.0450)	-
3j	Unpaired Student's *t*-test	*Atrogin1* (0.0220)<br>*Foxo3* (0.0082)	-
3k	Unpaired Student's *t*-test	*Il-6* (0.0440)	-
4b,c	Unpaired Student's *t*-test	4b (0.2561), 4c (0.0160)	-
4d	Two-way ANOVA + Šídák	*Atrogin1*<br>Sham (0.9991), Botox (0.0398)<br>*Murf1*<br>Sham (0.9973), Botox (0.0211)	DS109_B11 x BoNT-A<br><br>Atrogin1 (0.0788)<br>Murf1 (0.0495)
4f	Two-way ANOVA + Šídák	Atrogin1<br>Sham (0.3469), Botox (0.0009)<br>Murf1<br>Sham (0.8578), Botox (0.0225)	DS109_B11 x BoNT-A<br><br>Atrogin1 (0.0168)<br>Murf1 (0.0867)
4h,i	Two-way ANOVA + Šídák	Average CSA<br>Sham (0.7639), Botox (0.0467)<br><br>Distribution 4000-6000<br>Veh+Sham vs Veh+Botox (0.0185)<br>DS109+Sham vs DS109+Botox (0.2974)	DS109_B11 x BoNT-A<br><br>Average CSA (0.1700)<br><br>Distribution 4000-6000<br>(0.0103)

Figure	Statistical test	Post-hoc or t-test (P value)	Interaction P value
S2b	Unpaired Student's *t*-test	S2b (0.0494)	-
S2c	Unpaired Student's *t*-test	Grip strength<br>Week 0 (0.1624)<br>Week 6 (0.0017)	-
S2d	Unpaired Student's *t*-test	Treadmill<br>Time to exhaustion (0.0050)<br>Running distance (0.0050)	-
S3a	Unpaired Student's *t*-test	Atp5a (0.0017)<br>Uqcr2 (0.0004)<br>Mtco1 (0.0064)<br>Sdhb (0.0049)<br>Ndufb88 (0.0010)	-

Supplementary Table S3

Supplementary Table S3. Detailed statistical results for Supplementary Figures S2 and S3.

Supplementary Table S4

Gene	Forward (5'→3')	Reverse (5'→3')
Gapdh	AGG TCG GTG TGA ACG GAT TTG	TGT AGA CCA TGT AGT TGA GGT CA
36B4	GTC CTA GAC CAG TGT TCTGAG C	GCT TCG TGT TCA CCA AGG AGG A
Actin	GGC TGT ATT CCC CTC CAT	CCA GTT GGT AAC AAT GCC ATG
Myh3	AAA AGG CCA TCA CTG ACG C	CAG CTC TCT GAT CCG TGT CTC
MyoG	CTA CAG GCC TTG CTC AGC TC	ACG ATG GAC GTA AGG GAG TG
Mef2c	TCC ATC AGC CAT TTC ACC AA	GTT ACA GAG CCG AGG TGG AG
Mymx	GAC CAC TCC CAG AGG AAG GA	GGA CCG ACG CCT GGA CTA AC
Fbxo32	ACA AGG GAA GTA CGA AGG AGC G	GGC AGT CGA GAA GTC CAG TC
Trim63	GTG TGA GGT GCC TAC TTG CTC	GCT CAG TCT TCT GTC CTT GGA
Foxo3	CTG GGG GAA CCT GTC CTA TG	TCA TTC TGA ACG CGC ATG AAG
Ndufs1	AGG ATA TGT TCG CAC AAC TGG	TCA TGG TAA CAG AAT CGA GGG A
Cpt1b	CCC ATG TGC TCC TAC CAG AT	CCT TGA AGA AGC GAC CTT TG
HK2	CCG CCG TGG TGG ACA AGA TA	AGC AGT GAT GAG AGC CGC TC
Il-6	TTC AGG TGT CAA AGA CCA TCG C	CAG GTT TCA CCC TTC AGT TCC C

Supplementary Table S4. List of all primer sequences used for qRT-PCR.
